# Standardised Competency-Based Training of Medical Doctors and Associate Clinicians in Inguinal Repair with Mesh in Sierra Leone

**DOI:** 10.1007/s00268-023-07095-1

**Published:** 2023-07-14

**Authors:** Thomas Ashley, Hannah F. Ashley, Andreas Wladis, Pär Nordin, Michael Ohene-Yeboah, Rimantas Rukas, Vytautas Lipnickas, Isaac O. Smalle, Kristina Holm, Herta Kalsi, Juuli Palmu, Foday Sahr, Jessica H. Beard, Jenny Löfgren, Håkon A. Bolkan, Alex J. van Duinen

**Affiliations:** 1 Department of Surgery, Connaught Hospital, Freetown, Sierra Leone; 2 Upper Eden Medical Practice, Cumbria, UK; 3 Department of Biomedical and Clinical Sciences, Linköping University, Linköping, Sweden; 4 Department of Surgical and Perioperative Sciences, Umeå University, Umeå, Sweden; 5 Department of Surgery, University of Ghana Medical School, Korle Bu, Accra, Ghana; 6 Department of Surgery, St Olav’s Hospital, Trondheim University Hospital, Trondheim, Orkdal, Norway; 7 Department of Abdominal and Oncological Surgery, Vilnius University Hospital Santaros Klinikos, Vilnius, Lithuania; 8 Department of Anaesthesia and Intensive Care, Mälarsjukhuset, Eskilstuna, Sweden; 9 Department of Surgery, Capio St Görans Hospital, Stockholm, Sweden; 10 Department of Anaesthesiology and Intensive Care, Örebro University Hospital, Örebro, Sweden; 11 College of Medicine and Allied Health Sciences (COMAHS), Freetown, Sierra Leone; 12 Joint Medical Unit (JMU), Republic of Sierra Leone Armed Forces (RSLAF), Freetown, Sierra Leone; 13 Department of Surgery, Lewis Katz School of Medicine at Temple University, Philadelphia, PA USA; 14 Department of Molecular Medicine and Surgery, Karolinska Institutet, Solna, Sweden; 15 Institute of Nursing and Public Health, Norwegian University of Science and Technology (NTNU), Postboks 8905, 7491 Trondheim, Norway

## Abstract

**Introduction:**

In low-income settings, there is a high unmet need for hernia surgery, and most procedures are performed with tissue repair techniques. In preparation for a randomized clinical trial, medical doctors and associate clinicians received a short-course competency-based training on inguinal hernia repair with mesh under local anaesthesia. The aim of this study was to evaluate feasibility, safety and effectiveness of the training.

**Methods:**

All trainees received a one-day theoretical module on mesh hernia repair under local anaesthesia followed by hands-on training. Performance was assessed using the American College of Surgeon’s Groin Hernia Operative Performance Rating System. Patients were followed up two weeks and one year after surgery. Outcomes of the patients operated on during the training trial were compared to the 229 trial patients operated on after the training.

**Results:**

During three surgical camps, seven medical doctors and six associate clinicians were trained. In total, 129 patients were operated on as part of the training. Of the 13 trainees, 11 reached proficiency. Patients in the training group had more wound infections after two weeks (8.5% versus 3.1%; *p* = 0.041). There was no difference in recurrence and mortality after one year, and none of the deaths were attributed to the surgery.

**Discussion and conclusion:**

Mesh repair is the international standard for inguinal hernia repair worldwide. Nevertheless, this is not widely accessible in low-income settings. This study has demonstrated that short-course intensive hands-on training of MDs and ACs in mesh hernia repair is effective and safe.

*Trial Registration*: International Clinical Trial Registry ISRCTN63478884.

**Supplementary Information:**

The online version contains supplementary material available at 10.1007/s00268-023-07095-1.

## Introduction

In Sub-Saharan Africa, the estimated need for inguinal hernia repair is 204 to 210 per 100,000 population [1]. In Ghana, the estimated annual incidence of symptomatic inguinal hernia is 210 per 100,000 population, and a study from Sierra Leone has documented a prevalence of untreated groin hernia at 2.5% [2, 3]. Although inguinal hernia repair is among the most common surgical procedures performed, accounting for up to 16.0% of all surgeries in Sierra Leone, the met need remains low [4]. Similarly in Ghana, Tanzania and Uganda, the surgical backlog translates into millions of people living with an inguinal hernia [3, 5, 6]. Inadequate access to inguinal hernia repair in low-income settings leads to avoidable morbidity, mortality and economic loss [7].

In sub-Saharan Africa, inguinal hernias are usually repaired using tissue techniques due to the lack of availability of affordable mesh and limited training in mesh repair techniques for surgical providers [8]. Using mesh to achieve a tension-free inguinal hernia repair significantly reduces the risk of hernia recurrence and has been the preferred method in high-income countries for decades [9]. Mesh has been shown to be safe and effective in different settings in sub-Saharan Africa [10, 11]. The implementation of this technique in low resource settings would be a way to improve quality of inguinal hernia surgery.

In Sierra Leone, inguinal hernia repair is performed by surgeons, medical doctors (MDs) with no formal surgical training and by associate clinicians (ACs) who have an educational level between that of a nurse and an MD [12]. A single-blind, noninferiority randomized clinical trial including adult, healthy men with primary inguinal hernia found that surgical task-sharing with ACs in elective groin hernia repair was safe and effective [13].

Standardized training for inguinal hernia repair with mesh is not required for Sierra Leonean MDs and ACs but can be a way to ensure quality of both training and surgical care delivery [14]. The aim of this study was to evaluate safety of a standardized short-course competency-based training for groin hernia mesh repair by comparing clinical outcomes between operations performed during training and those performed immediately after by the providers that reached proficiency. The latter constitutes results from a previously published intervention trial [13]. In addition, we aimed to compare training performance between MDs and ACs.

## Methods

### Study setting

Sierra Leone is a low-income country that ranks 181 of 189 countries on the Human Development Index [15]. The country suffers major limitations in the health-care system, and the unmet need for surgery is immense. The study was conducted at Kamakwie Wesleyan Hospital, a first-level hospital located in the north-western region of Sierra Leone. This hospital provides basic obstetric, paediatric, general medical and surgical care for its 200,000-catchment population.

### Study participants

Both trainees and patients were study participants. The trainees were MDs and ACs. The MDs had all completed at least one year of internship including at least six months of general surgery, and they were not enrolled into a surgical residency program at the time of the study. The ACs had graduated from the CapaCare training programme, a two-year surgical training programme followed by a one-year internship [16]. Both MDs and ACs were required to be independently performing hernia repair using sutured techniques to participate in this training.

The patients were above 18 years, healthy, American Society of Anaesthesiologists physical status (ASA) score 1 and 2, men with reducible primary inguinal hernia who were able to give informed consent. Substance or alcohol abuse and known coagulation disorder were exclusion criteria.

The trainers were five senior consultant general surgeons from Ghana, Norway Lithuania, and Sweden (Table 1). The training was organised in three camps and as the preparation training for a larger study comparing outcomes of mesh repair for inguinal hernias performed by MDs and ACs [13]. Ethical approval was obtained from the Sierra Leone Ethics and Scientific Committee (Identification code—Dr Thomas Ashley 22.05.2017I) and registered at the International Clinical Trial Registry (ISRCTN63478884).

**Table 1:** Trainee characteristics

Characteristic	Value (*n* = 13)
Age, median, (IQR) in years	33 (30–34)
Sex, *n*, (%)	
Male	10 (77%)
Female	3 (23%)
Training level, *n*, (%)	
Medical doctor	7 (54%)
Associate clinician	6 (46%)
Place of work, *n*, (%)	
First-level hospital	3 (23%)
Tertiary Hospital	10 (77%)
Surgical experience*, median, (IQR) in years	3.5 (1–4)
Experience tissue repair, median, (IQR) number	
Assisted tissue repair	25 (10–40)
Performed tissue repair	55 (11–90)
Experience mesh repair, median, (IQR) number	
Assisted mesh repair	5.5 (2–10)
Performed mesh repair	3 (0–5)
Total experience inguinal hernia repair, median, (IQR) number	102 (54–300)

^*^Time after graduation

*CI* confidence interval; *IQR* interquartile range

### Outcome measures

The primary outcomes for this study were hernia recurrence and mortality within 1 year after the operation. Other outcomes were operation time, surgical site infections after 2 weeks and patient satisfaction and chronic pain after 1 year. Chronic pain was assessed with the Inguinal Pain Questionnaire (IPQ) [17]. In addition, the proportion of trainees reaching proficiency in mesh inguinal hernia repair and the number of surgeries they required to reach this were assessed.

### Surgical procedure

In this study, the MDs and ACs were trained to perform an anterior inguinal hernia mesh repair according to Lichtenstein under local anaesthesia method which was followed stepwise [18, 19]. A light-weight commercial polypropylene mesh produced by Medtronic was used for all patients. Prior to the surgery, prophylactic antibiotic (Flucloxacillin 1.5 g) was given to each patient within 4 h of the procedure. If local anaesthesia was inadequate, general anaesthesia was applied using Ketamine.

### The training

The training consisted of two modules; one theoretical and one practical with supervised, hands-on surgery. The theoretical module lasted one day and was delivered by interactive, PowerPoint supported presentations developed by The Ghana Hernia Society. The content included anatomy of the groin, step-by-step explanation of local anaesthetic infiltration and the Lichtenstein method of inguinal hernia repair.

During the practical module, each trainee observed, assisted and performed a number of inguinal hernia repairs with mesh. Trainees first assisted the trainer in at least one operation, followed by the trainee being the principal surgeon assisted by the trainer and then trainees assisting each other. Paired trainees were allowed to observe operations being performed by the other trainee when their counterpart was being assisted by a trainer. All training operations were done with at least one consultant surgeon present in theatre. To ensure patient safety, surgeon trainers took over operations if they thought the case was too difficult for the trainee. These cases are included in this study as training procedures but not as assessment procedures before qualification.

The performance of each trainee was assessed after each surgery. The assessment was verbal and also delivered in a structured, written form based on the American College of Surgeon’s Groin Hernia Operative Performance Rating System [20]. In this rating system, scores range from a minimum of 1 to a maximum best score of 5. Proficiency was determined as at least twice an overall score of 4 or 5 observed by two consultant surgeons separately. Thereafter, the trainee was allowed to operate on patients independently. This system of assessment has been validated and used in various low resource settings [21]. In addition to the operative performance rating system, trainers considered patient safety in the hands of the trainees.

### Statistical analysis

Descriptive statistics were used to present trainer, trainee and patient characteristics. Student’s *t* test was used for comparison of numerical means and Fisher’s exact test to compare categorical data. *p* < 0.050 was considered statistically significant. Median and inter-quartile range (IQR) were used to present non-normal distributed data. Statistical analyses were carried out with STATA 16.0 (StataCorp LLC, College Station, USA).

## Results

The study was carried out during three surgical camps in October 2017, November 2017 and February 2018. Thirteen Sierra Leonean MDs (*n* = 7) and ACs (*n* = 6) participated as trainees (Table 1). The trainees had on average 2.8 years of clinical experience after graduation, and all of them had no or limited experience with performing mesh repair for inguinal hernias.

**Fig. 1:**
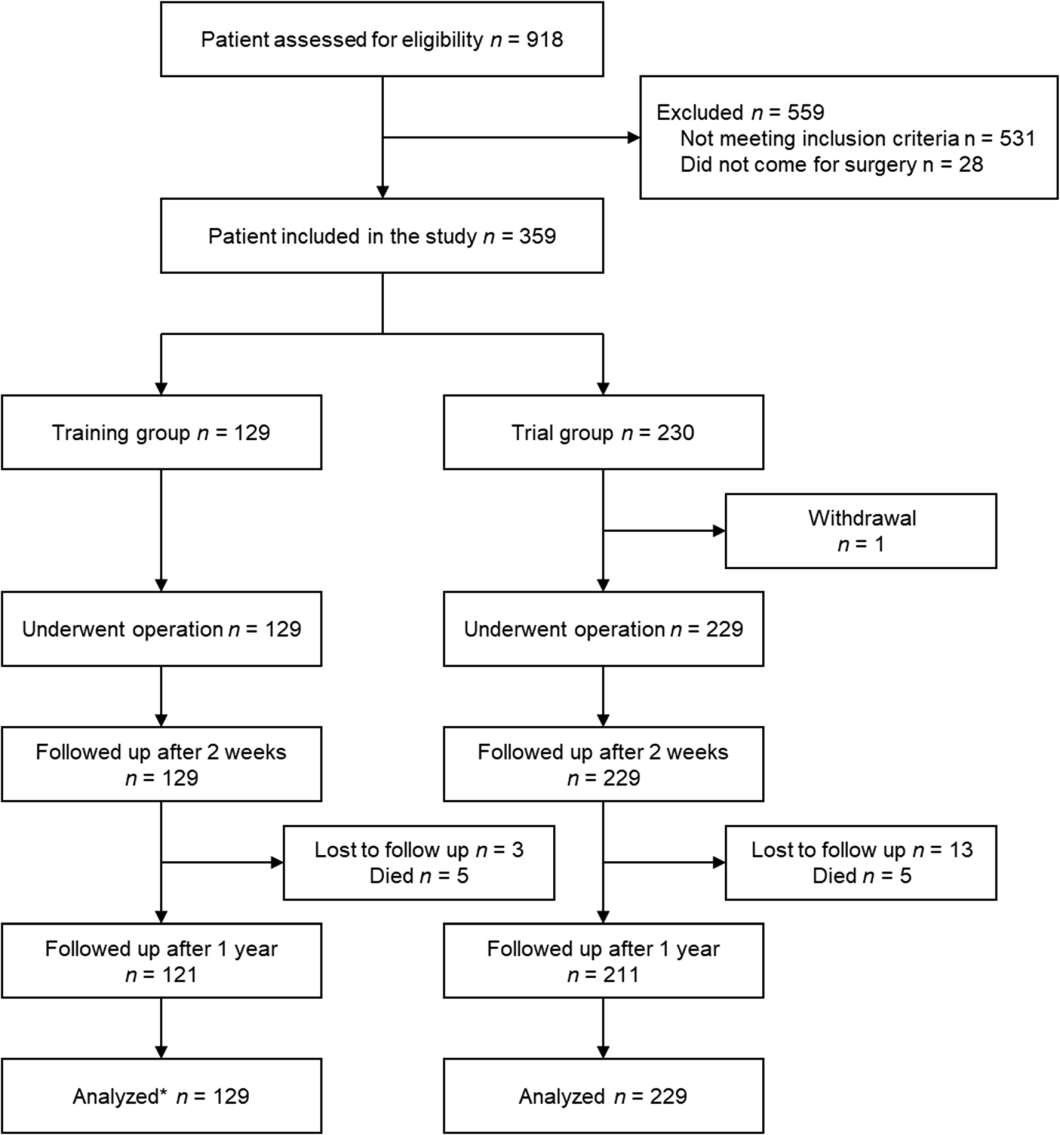
Study flowchart. *Of the 129 training patients analysed, 72 procedures performed for qualification

A total of 918 patients were screened for the training and trial combined (Fig. 1). Of those, 531 did not meet the inclusion criteria, and 28 did not come for surgery leaving 359 being included, 129 in the training study and 230 in the subsequent trial. Of the 129 training surgeries, 72 (55.8%) were performed to reach qualification to participate in the trial. The other 57 (44.2%) procedures were part of the training with the aim to demonstrate techniques by the trainers and to practice after qualification for the trainees.

Of the 13 trainees, 11 reached proficiency. The two who did not reach proficiency were both MDs (Table 2). The trainees were assistant surgeon for an average of 1.1 and 2.2 cases and lead surgeon for an average of 5.8 and 5.2 for MDs and ACs, respectively. Difficulty and operation time were similar for both groups.

**Table 2:** Surgeries before qualification performed by medical doctors and associate clinicians

Outcomes	Medical doctors (*n* = 7)	Associate clinicians (*n* = 6)	*p*-value
Procedures as lead surgeon	*n* = 41	*n* = 31	
Number, mean, (95% CI)	5.8 (4.2–7.5)	5.2 (2.8–7.5)	0.546
Operation time, mean, (95% CI)	67 (61–74)	63 (57–69)	0.263
Difficulty score, mean, (95% CI)	2.3 (1.9–2.6)	2.3 (1.8–2.8)	0.915
Procedures as assistant	*n* = 8	*n* = 13	
Number, mean, (95% CI)	1.1 (0.2–2.1)	2.2 (0.5–3.8)	0.196
Procedures before qualification*, mean, (95% CI)	7.4 (4.4–10.4)	7.3 (5.0–9.6)	0.962

^*^Two medical doctors did not reach proficiency and therefore not included in this analysis

*CI* confidence interval

Table 3 shows the comparison of patient-level outcomes between the training and trial group. In the training group, the self-assessed health score was lower compared to the trial group (74.5% versus 78.2%; *p* = 0.023). The operation time was almost 10 min longer in the training group compared to the trial group (66.4 versus 57.2 min; *p* < 0.001).

**Table 3:** Patient and perioperative characteristics

Characteristic	Training (*n* = 129)	Trial (*n* = 229)	*p*-value
Age, mean, (95% CI) in years	44.4 (41.7–47.1)	42.8 (41.1–44.6)	0.314
BMI, mean, (95% CI)	22.1 (21.3–23.0)	21.5 (21.0–22.0)	0.176
ASA classification, *n*, (%)			
ASA I	58 (45.0)	103 (45.0)	1.000
ASA II	71 (55.0)	126 (55.0)	
Current smoking, *n*, (%)	54 (41.9)	100 (43.0)	0.824
Previous smoking, *n*, (%)	24 (18.6)	35 (15.3)	0.459
Scrotal hernia, *n*, (%)	63 (48.8)	117 (51.1)	0.741
IPQ score, mean, (95% CI)	3.2 (2.9–3.5)	3.0 (2.8–3.2)	0.252
Self-assessed health score, mean, (95% CI)	74.5 (71.9–77.1)	78.2 (76.2–80.1)	*0.026*
Perioperative characteristics			
Operation time, min, (95% CI)	66.4 (62.3–70.4)	57.2 (54.9–59.4)	*0.000*
Local anaesthesia only, *n*, (%)	126 (97.7)	228 (99.6%)	0.135

*ASA* American Society of Anaesthesiologists; *BMI* body mass index (calculated as weight in kilograms divided by height in meters squared); *CI* confidence interval; *IPQ* Inguinal Pain Questionnaire

Patients in the training group had at the two-week follow-up more than twice as many wound infections requiring antibiotics compared to those in the trial group (8.5% versus 3.1%; *p* = 0.041). There was no difference in recurrence after one year between the training and the trial group (5.4% versus 3.5%; *p* = 0.423) (Table 4). The mortality at one-year follow-up was with respectively 2.2% and 3.9% not significantly different (*p* = 0.509). None of the deaths in either group appeared to be related to the surgery performed, and all occurred more than 6 months after the hernia repair (Table S1).

**Table 4:** Training versus trial patients’ outcomes after 2 weeks and 1 year

Outcomes	Training (*n* = 129)	Trial (*n* = 229)	*p*-value
Outcome after 2 weeks			
Any complication, *n*, (%)	40 (31.0)	57 (24.9)	0.218
Pain more than expected, *n*, (%)	3 (2.3)	3 (1.3)	0.671
Impaired wound healing, *n*, (%)	13 (10.8)	20 (8.7)	0.705
Wound infection requiring antibiotics, *n*, (%)	11 (8.5)	7 (3.1)	*0.041*
Hematoma/seroma, *n*, (%)	12 (9.3)	17 (7.4)	0.549
Reoperation, *n*, (%)	1* (0.8)	3† (1.3)	1.000
Outcome after 1 year			
Recurrence	7 (5.4)	8 (3.5)	0.423
Death	5 (3.9)	5 (2.2)	0.509
Satisfied with the result	118 (91.5)	207 (90.4)	0.360
IPQ score, mean, (95% CI)	1.2 (1.1–1.4)	1.2 (1.1–1.3)	0.925
IPQ score change, mean, (95% CI)	− 1.9 (− 2.2– − 1.6)	− 1.7 (− 1.9– − 1.5)	0.205
Self-assessed health score, mean, (95% CI)	88.2 (86.3–90.0)	88.5 (87.1–90.2)	0.822
Health score change, mean, (95% CI)	13.3 (10.3–16.3)	9.9 (7.4–12.4)	0.094

^*^Incision and drainage

^†^Incision and drainage, evacuation of a hematoma and reoperation for early recurrence

*CI* confidence interval; *IPQ* Inguinal Pain Questionnaire

## Discussion

This study demonstrates that an intensive hands-on, competency-based short-course training in mesh hernia repair for MDs and ACs with experience in tissue hernia repair is feasible and safe. Two MD trainees did not reach proficiency, and the remaining trainees reached proficiency after an average of between 5 and 6 procedures as the lead surgeon.

Open anterior mesh inguinal hernia repair is the gold standard technique for adult men globally. Currently, this is not standard practice in Sierra Leone due to cost and limited availability of mesh along with lack of widespread training in this technique. The situation is comparable to other sub-Saharan countries [22, 23]. Ensuring supply of mesh at low cost along with the implementation of a short course in the technique for surgical providers would be a way to rapidly improve the quality of groin hernia surgery in Sierra Leone. The MDs and ACs that participated in this study had already substantial experience with non-mesh inguinal hernia repairs and were familiar with the complex anatomy of the groin. As one of the reasons not to perform mesh-repair for groin hernias is the lack of experience, a short-course competency-based training programme can take away this barrier and contribute to quality improvement in surgical care [22].

Patient outcomes were favourable, with an overall recurrence rate of 3.9% after one year in the training group. This is much lower than the 13.1% recurrence rate that was identified from a nation-wide population study from Sierra Leone. However, most of those surgeries were done without mesh and with a longer follow-up time [2]. A previous study in Ghana demonstrated a recurrence rate of 1.8% after one year when the procedures were performed by general surgeons and MDs [24].

The surgical site infection rate in the training group was more than double compared to the trial group. In previous research, an association between increased operating time and surgical site infections has been described [25]. Another factor that might have contributed to a lower number of wound infections in the trial group is that perioperative routines were more established, and collaboration in the surgical teams had improved due to the obtained experience during the training surgeries. The operation time in the training group was 66.4 min, 9.2 min longer than in the trial group, as expected. Similar findings have been described by van Kesteren et al. analysing operation time for hernia surgeries observing a reduction in average operating time from 80 min in the first five procedures until below 50 min after 30 procedures [26]. In order to reduce operating time and risk for wound infections, other additional training modalities can be considered in the future such as the use of simulation models [27].

We found the one-year mortality in the training group was more than double that of the trial group (4.7% versus 2.2%). For all patients that died, the cause of death was recorded based on interviews. All deaths occurred more than 6 months after surgery, and none of the deaths could be attributed to the hernia surgery. Taking all this into account, we conclude that a short competency-based training is a safe and effective way to train MDs and ACs how to perform a mesh inguinal hernia repair under local anaesthesia in adult men in this low resource setting.

In order to reach a recurrence rate for groin hernia mesh repair of about 1% as described in high-income settings, more efforts are needed [28, 29]. Possible ways to enhance further reduction in recurrences might be to assess and improve preoperative nutritional status and encourage smoking cessation to minimize postoperative complications [30, 31]. Another proven strategy to improve quality and reduce complications is institutional certification by standardizing surgical management [32]. Even though these strategies have been proven to be effective in high-income settings, further research is needed to assess effectiveness in low resource settings.

### Strengths and limitations

This study has several strengths. The internal validity is high due to the rigorous oversight of the primary investigator (TA). It was implemented in a rural district hospital, where most hernia surgeries takes place in Sierra Leone; therefore, its results would be generalizable to other similar hospitals [4, 33]. The one-year follow-up period of the patients provides an extra strength as assessed proficiency of the trainee should be confirmed by beneficial patient outcomes.

The results of this study provide evidence of the feasibility and safety of training non-specialist surgical providers in a procedure that is much needed in the context of the human resource crisis of low-income countries such as Sierra Leone. A limitation of this study is the fact that the trainees had pre-training knowledge and experience on how to perform tissue inguinal hernia repairs. Recruitment of MDs was challenging due to their limited number and the fact that they are often the only surgical-skilled doctor at their hospital, and therefore not easily able to take time away from their jobs.

## Conclusion

Mesh repair is the standard method for open repair of inguinal hernia worldwide. Nevertheless, this technique is not widely accessible in low-income settings. This study has demonstrated that short-course competency-based training of MDs and ACs in mesh hernia repair is feasible, effective and safe. However, the cost and accessibility of the mesh and costs and capacity of training surgical providers in mesh repair remain important barriers to upscale this preferred technique. Only with the combined efforts of patient groups, service providers, policy makers and donors, it will be possible to reduce the burden of untreated inguinal hernias in low-income settings.

## Supplementary Information

Below is the link to the electronic supplementary material.

Supplementary file1 (DOCX 21 kb)
