# Supplementary material for: Standardised Competency-Based Training of Medical Doctors and Associate Clinicians in Inguinal Repair with Mesh in Sierra Leone
Source: World J Surg. 2023 Jul 14;47(10):2330–7. doi: 10.1007/s00268-023-07095-1 (PMC10474210; doi:10.1007/s00268-023-07095-1)
Supplement: Supplementary file 1 — Supplementary file1 (DOCX 21 kb) [file 268_2023_7095_MOESM1_ESM.docx]

# Table S1. Mortality in training and trial group

|  | Group | Camp | Age  (years) | ASA | Operation time (min.) | Time after surgery (mnth.) | Cause of death |
| --- | --- | --- | --- | --- | --- | --- | --- |
| 1 | training | II | 21 | 2 | 57 | 6 | Right lower limb swelling |
| 2 | training | II | 67 | 2 | 58 | 15 | Chronic illness |
| 3 | training | II | 75 | 1 | 35 | 8 | Acute disease unrelated to hernia, ill after surgery ill for one day and died |
| 4 | training | II | 37 | 1 | 42 | 13 | Respiratory disease |
| 5 | training | III | 37 | 2 | 55 | 13 | Hypertension |
| 6 | trial | I | 50 | 2 | 48 | 13 | Snake bite |
| 7 | trial | II | 36 | 2 | 34 | 6 | No information available |
| 8 | trial | II | 58 | 1 | 51 | 9 | Admitted with acute abdominal pain, died after 2 days later. Suspected perforated ulcer, did not receive surgical treatment |
| 9 | trial | II | 54 | 2 | 45 | 9 | Chronic abdominal pain that had started already prior to the hernia surgery after fall from palm tree |
| 10 | trial | III | 80 | 2 | 56 | 6 | Hypertension |

ASA = American Society of Anaesthesiologists
